# Assessment of High-Resolution Melting Curve Analysis for *Leishmania* spp. Detection in Different Clinical Manifestations of Leishmaniasis in India

**DOI:** 10.3390/pathogens13090759

**Published:** 2024-09-04

**Authors:** Mudsser Azam, Saurabh Singh, Ratan Gupta, Mayank Mayank, Sushruta Kathuria, Shruti Sharma, V. Ramesh, Ruchi Singh

**Affiliations:** 1 ICMR-National Institute of Pathology, Safdarjung Hospital Campus, New Delhi 110029, India; mayanksangam8@gmail.com (M.M.); shrutigautam@rediffmail.com (S.S.); 2 Department of Dermatology, Venereology, Leprology, All India Institute of Medical Sciences, Jodhpur 342005, India; saurabhdoc@yahoo.co.in; 3 VMMC and Safdarjung Hospital, New Delhi 110029, India; ratangupta100@yahoo.com (R.G.); drsushruta@gmail.com (S.K.); 4 Department of Dermatology and STD, ESIC Medical College & Hospital Faridabad, Faridabad 121012, India; weramesh@gmail.com

**Keywords:** leishmaniasis, *Leishmania* species, high-resolution melting analysis, post-kala-azar leishmaniasis, cutaneous leishmaniasis, real-time polymerase chain reaction

## Abstract

The accurate diagnosis and identification of *Leishmania* species are crucial for the therapeutic selection and effective treatment of leishmaniasis. This study aims to develop and evaluate the use of high-resolution melting curve analysis (HRM)-PCR for *Leishmania* species identification causing visceral leishmaniasis (VL), post-kala-azar dermal leishmaniasis (PKDL) and cutaneous leishmaniasis (CL) in the Indian subcontinent. Two multi-copy targets (ITS-1 and 7SL-RNA genes) were selected, and an HRM-PCR assay was established using *L. donovani*, *L. major*, and *L. tropica* standard strain DNA. The assay was applied on 93 clinical samples with confirmed *Leishmania* infection, including VL (*n* = 30), PKDL (*n* = 50), and CL (*n* = 13) cases. The ITS-1 HRM-PCR assay detected as little as 0.01 pg of template DNA for *L. major* and up to 0.1 pg for *L. donovani* and *L. tropica*. The detection limit for the 7SL-RNA HRM-PCR was 1 pg for *L. major* and 10 pg for *L. donovani* and *L. tropica*. The ITS-1 HRM-PCR identified 68 out of 93 (73.11%) leishmaniasis cases, whereas 7SL-RNA HRM-PCR could only detect 18 out of 93 (19.35%) cases. A significant correlation was observed between the kDNA-based low Ct values and ITS-1 HRM-PCR positivity in the VL (*p* = 0.007), PKDL (*p* = 0.0002), and CL (*p* = 0.03) samples. The ITS-1 HRM-PCR assay could identify *Leishmania* spp. causing different clinical forms of leishmaniasis in the Indian subcontinent, providing rapid and accurate results that can guide clinical management and treatment decisions.

## 1. Introduction

*Leishmania* is a genus of hemoflagellate obligate intracellular parasitic protozoa that causes a spectrum of diseases collectively known as leishmaniases. The species under the genus *Leishmania* are responsible for causing visceral and dermal forms of leishmaniases. Visceral leishmaniasis (VL) is the most severe form and is a life-threatening condition. Cutaneous leishmaniasis (CL) affects the skin, while mucocutaneous leishmaniasis (MCL) manifests on both the skin and mucous membranes. Around 20 species cause infections in humans, whereby the distribution of *Leishmania* species varies geographically, and each species may be associated with one or more clinical manifestations of the disease [1,2].

In India, three major clinical manifestations of leishmaniasis—VL and two dermal forms, post-kala-azar dermal leishmaniasis (PKDL) and CL—have been documented and are caused by different parasite species [3]. VL, also known as kala-azar, is caused by *Leishmania donovani* and is transmitted anthroponotically by the bite of an infected female sandfly, *Phlebotomus argentipes*. VL is endemic in northern and eastern states of India, including Bihar, Jharkhand, Uttar Pradesh, and West Bengal. India, along with the other five endemic countries, i.e., Brazil, Ethiopia, Eritrea, Kenya, and Sudan, accounted for 79% of total VL cases reported globally [4]. PKDL is a dermal sequel that occurs in approximately 5–10% of treated cases of VL caused by *L. donovani*. It usually develops 1–3 years after the completion of therapy [3,5]. Conversely, CL is caused by *L. major* in Jammu and *L. tropica* in the hot, arid western region of Rajasthan [6].

Various clinical conditions can arise from different *Leishmania* species and even from the same species with significant genetic variations [7,8,9]. The discrete chromosomal and gene copy number variations or single nucleotide polymorphisms (SNPs) as underlying genetic alterations give rise to atypical variants. For instance, VL can be caused not only by the usual viscerotropic *L. donovani* species complex, including *L. infantum* and *L. donovani*, but also by *L. tropica*. Similarly, the *L. donovani* complex species can cause CL in addition to the typical dermotropic species, such as *L. major* and *L. tropica*. There have been a few sporadic and imported cases of leishmaniasis reported in non-endemic parts of India, including Assam, Delhi, Kerala, Sikkim, Tamil Nadu, and Uttarakhand [3]. Exceptionally, *L. tropica* has also been isolated from VL cases in the Indian subcontinent [6]. Additionally, *L. donovani*, responsible for VL (*L. donovani* zymodeme MON2), has also been reported from CL cases from Himachal Pradesh (similar to *L. donovani* zymodeme MON37) and Kerala, India [6,10,11,12].

*Leishmania* species are differentiated on the basis of variations in the genome. Sequence variations among the conserved regions are exploited for species identification. While multi-locus enzyme electrophoresis (MLEE) is considered the gold standard, its reliance on parasite isolation and culture limits its application. Several other techniques have been employed for *Leishmania* species identification, such as selective target amplification and sequencing [13], restriction fragment length polymorphism (RFLP) [14], multi-locus sequencing typing (MLST), and mass spectrometry [15]. Microsatellites, kinetoplastid DNA (kDNA), telomeric sequences, internal transcribed spacer-1 (ITS-1), heat shock proteins like HSP70 and HSP60, and genes involved in metabolic processes like mannose phosphate isomerase and 6-phosphogluconate dehydrogenase are typically used as amplification targets in these techniques. Certain markers, in particular, kDNA, ITS-1, and HSP70, have been the most extensively exploited molecular targets because of their abundance in copies throughout the *Leishmania* genome, which raises the sensitivity of the test [15]. Some of these methods have limitations such as time-consuming post PCR procedures, complex data interpretation, low sensitivity, high cost, and potential for cross-reactivity.

High-resolution melting curve (HRM) analysis is a valuable tool for identifying genetic variations based on differences in melting temperatures following PCR. It is a well-established molecular technique that has demonstrated high confidence and reproducibility in identifying pathogens at the species level. The advancement in real-time PCR instrumentation with precise temperature ramp control and advanced data capture capabilities, combined with dsDNA-binding dyes, allows for fine data analysis and manipulation using specifically designed software. Melting curves are generated by plotting the decrease in fluorescence during melting against the steady rate of temperature increase. The HRM-PCR technique has several advantages over traditional methods, such as high sensitivity, rapid turnaround time, cost-effectiveness, no cross contamination, and the ability to distinguish the genotype indicating the distinct pathogen [16,17,18]. Genes or targets with high copy numbers increase the sensitivity of *Leishmania* parasite detection in HRM-PCR assays. Some of the markers that have been employed for species identification in *Leishmaniasis* include the heat shock protein 70 gene (*HSP70*), *ITS-1* sequences, the *7SL-RNA* gene, the *LACK* gene, and the amino acid permease gene [19,20,21,22,23,24].

The precise diagnosis and identification of the *Leishmania* species are vital for selecting the proper treatment and ensuring the effective management of leishmaniasis. Various HRM-based assays have been developed earlier for parasite species identification using reference strains of cultured *Leishmania* parasites. These assays have been applied and validated in human clinical samples, infected sandflies, and reservoir animals, revealing the high sensitivity and specificity of HRM-PCR in identifying different *Leishmania* species, including *L. donovani*, *L. infantum*, *L. major*, and *L. tropica* in both the Old World and New World [25]. However, its use in Old World settings, such as India, has not been extensively explored. This study aimed to select the appropriate target and to develop and assess the application of the HRM-PCR assay for detecting *Leishmania* spp., which yields various clinical manifestations of leishmaniasis in India.

## 2. Materials and Methods

### 2.1. Standard Parasite Strains; Study Population and Samples

The HRM-PCR assay was established using the genomic DNA of the standard strains of *L. donovani*, *L. major*, and *L. tropica* listed in Table 1 along with ITS-1 sequence accession numbers. The present study included human clinical samples from clinically diagnosed cases of leishmaniasis, including cases of VL, PKDL, and CL, who reported to Safdarjung Hospital, New Delhi; ESIC Medical College and Hospital, Faridabad; and All India Institute of Medical Sciences (AIIMS), Jodhpur, India. Blood samples from VL patients and skin biopsies from PKDL and CL patients were collected. The clinically diagnosed cases confirmed by quantitative real-time PCR (q-PCR) were included for species identification using target amplification by real-time PCR and HRM analysis (HRM-PCR).

### 2.2. q-PCR for Leishmania Parasite Estimation

Real-time PCR based on SYBR Green I using kDNA-based genus-specific primers was performed to quantify the parasite following the methods described earlier [26]. Briefly, DNA extraction was carried out using the DNeasy Blood & Tissue Kit (Qiagen, Hilden, Germany) following the manufacturer’s recommendations. PCR amplification and detection were performed using a BioRad CFX Opus 96 detection system (BioRad, Singapore). The PCR reaction (10 µL) consisted of 1X SYBR Green I PCR Master mix (Invitrogen, Europe), 200 nM each of forward and reverse primer, and 2 µL of isolated DNA. Cycling conditions included initial denaturation for 2 min at 95 °C, 40 cycles of denaturation for 10 s at 95 °C, and annealing for 30 s at 60 °C, followed by a melt curve.

### 2.3. Target Selection for HRM-PCR

**Figure 1:**
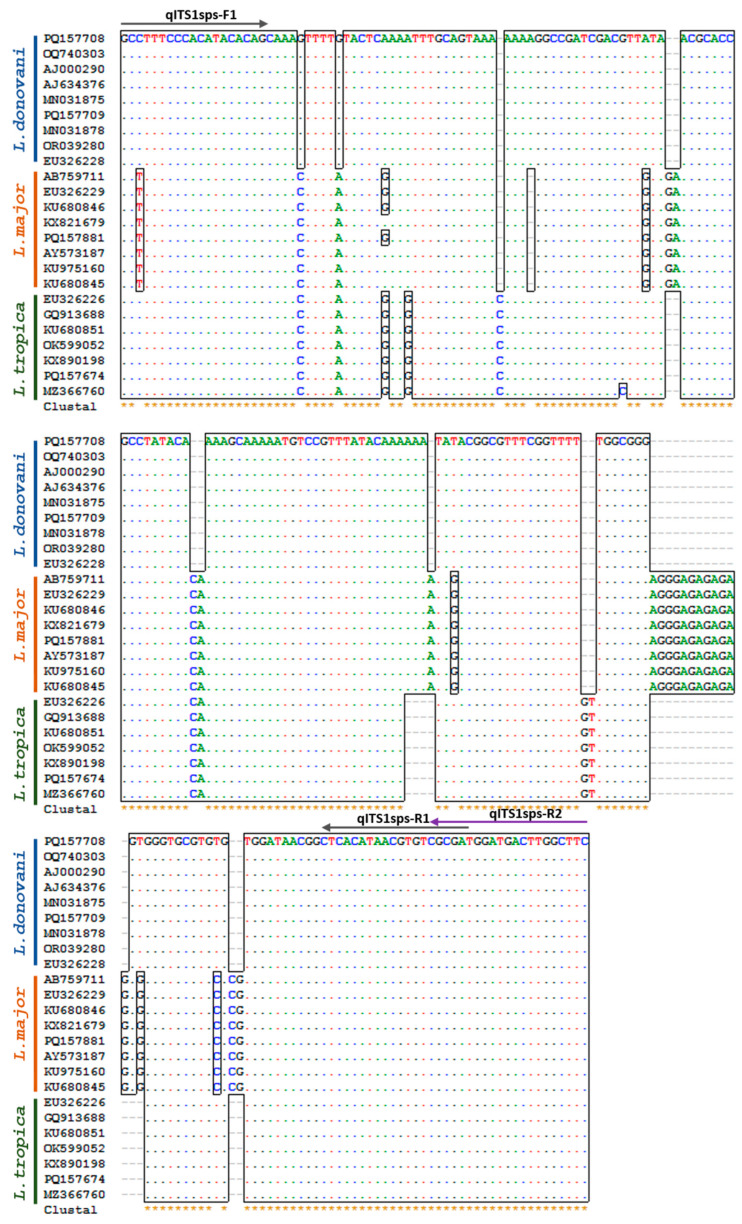
Alignment of ITS-1 gene fragment sequences from *Leishmania* species, i.e., *L. donovani*, *L. major*, and *L. tropica*, with an illustration of primers and sequence variation within the amplicon of HRM-PCR. ITS-1 gene: Internal Transcribed Spacer-1 rRNA gene.

A literature survey was conducted to investigate the targets used to identify the various *Leishmania* species that cause different manifestations in both human and non-human hosts. Two potential targets reported for the identification of *Leishmania* species among the Old World *Leishmania* strains were chosen for the identification of *Leishmania* species: the ITS-1 gene and the 7SL-RNA gene. These targets have shown high sensitivity in previous studies [19,27,28,29,30]. In silico research was performed, and multiple ITS nucleotide sequences for *L. donovani*, *L. major*, and *L. tropica* were downloaded (File S1_ITS sequences). The consensus sequences in the ITS region were selected for primer designing, as per the experimental design considerations for optimal HRM analysis [31], and two sets of primers were designed and assessed for the ITS-1 gene: qITS1sps-F1/R1 and qITS1sps-F1/R2 (a common forward primer and two different reverse primers for amplicons of different length). Multiple alignment of ITS-1 gene fragment sequences from the *Leishmania* species, i.e., *L. donovani*, *L. major,* and *L. tropica*, illustrating the selected primer regions and sequence variation within the amplicon of HRM-PCR, are shown in Figure 1. Primers previously used by Nasereddin and Jaffe were employed for the 7SL-RNA gene [30] (Table 2).

### 2.4. HRM-PCR Assay

PCR conditions for high-resolution melt curve analysis were standardized for primer concentrations (ranging from 100 nM to 500 nM for amplifying the ITS-1 gene as well as the 7SL-RNA gene) and annealing temperature (58 °C–63 °C). The final RT-PCR was set up in a CFX Opus 96 real-time PCR machine (Bio-Rad, Singapore) as follows: 10 µL reaction containing 2 µL of template DNA, 5 µL Precision Melt Supermix (Bio-Rad, Hercules, CA, USA), 300 nM of each forward and reverse primer, and sterile DNase/RNase-free water. Amplification conditions were set as follows: initial denaturation for 2 min at 95 °C; 40 cycles of denaturation for 10 s at 95 °C, annealing for 30 s at 60 °C, and extension for 30 s at 72 °C. For HRM analysis, duplex formation was performed at 70 °C and 95 °C for 1 min. The amplicon dissociation analysis was performed by capturing fluorescence signals in 0.2 °C intervals and holding for 10 s in each range of the melting curve (between 75 °C and 95 °C). The acquisition of fluorescence data and the construction of dissociation profiles were performed using Precision Melt Analysis^TM^ software v1.3 (BioRad). The normalized melt curves obtained for standard strains were analyzed for their melting profile. The 7SL-RNA HRM-PCR assay condition described earlier was attempted; however, it did not result in amplification [30].

### 2.5. Analytical Sensitivity and Specificity

The limit of detection (LOD) was estimated using 10-fold serial dilutions from 10 ng to 1 fg of DNA from standard strains—*L. donovani* (MHOM/IN/95/9515), *L. major* (MHOM/SN/74/SD), and *L. tropica* (MHOM/AF/87/RUP)—using the above standardized conditions. Each serial dilution prepared from the *Leishmania* standard strains was spiked with 40 ng of human DNA as background. PCR efficiency was evaluated using the threshold cycle (Ct) generated for the DNA template (in 10-fold serial dilutions) within the detection limits of each primer set for all three standard strains. Intra-assay reproducibility and changes in the melting temperature (Tm) of the ITS-1 amplicon in the HRM assay with respect to parasite load were observed by noting the average Tm and Ct values for the complete dilution series performed in two independent experiments.

The specificity of assay was evaluated using non-leishmania DNA templates from *Plasmodium falciparum* (MRA-327G), *Mycobacterium leprae* strain Br 4923 (NR-19351), *Toxoplasma gondii* strain GT1 (NR-20728), *E. coli* strain MG1655 (NR-2653), and non-infected healthy control human DNA samples (negative by RT-PCR, *n* = 15). *Leishmania* endemic locations are also inhabited by other *Trypanosoma* spp. An in silico analysis of ITS-1 gene sequences from *Trypanosoma* spp. (*T. cruzi* (AF362825), *T. evansi* (MN121259), *T. equiperdum* (LC386049), and *T. brucei* (MW364108)) was conducted to determine the specificity of the assay.

### 2.6. Validation of HRM-PCR on Leishmaniasis Patient Samples

To validate the developed HRM-PCR assay, 93 naturally infected human patient samples diagnosed by qPCR were tested. DNA extracted from the blood samples of VL patients (*n* = 30) and the tissue/slit aspirates of PKDL (*n* = 50) and CL (*n* = 13) patient samples were used as DNA templates for HRM-PCR following the standardized reaction conditions. The melting curves of patient DNA samples were compared against the curves for standard strains to identify the *Leishmania* species.

## 3. Results

### 3.1. HRM-PCR for Leishmania Species Identification

An HRM-PCR was established using the primer set for the ITS-1 gene and 7SL RNA gene targets based on reports for Old World *Leishmania* species identification. For the developed assays, optimum amplification was noted at 300 nM forward and reverse primer for the ITS-1 gene and 400 nM forward and reverse primer for the 7SL RNA gene, after annealing at 60 °C for 30 s and extension at 72 °C for 30 s using a DNA template from standard strains.

For the ITS-1 gene HRM-PCR (primer set qITS1sps-F1/R1), the melting temperature (Tm) noted for *L. donovani*, *L. major,* and *L. tropica* were 81.82 ± 0.46 °C, 83.3 ± 0.70 °C, and 82.4 ± 0.51 °C, respectively (Table 3).

**Figure 2:**
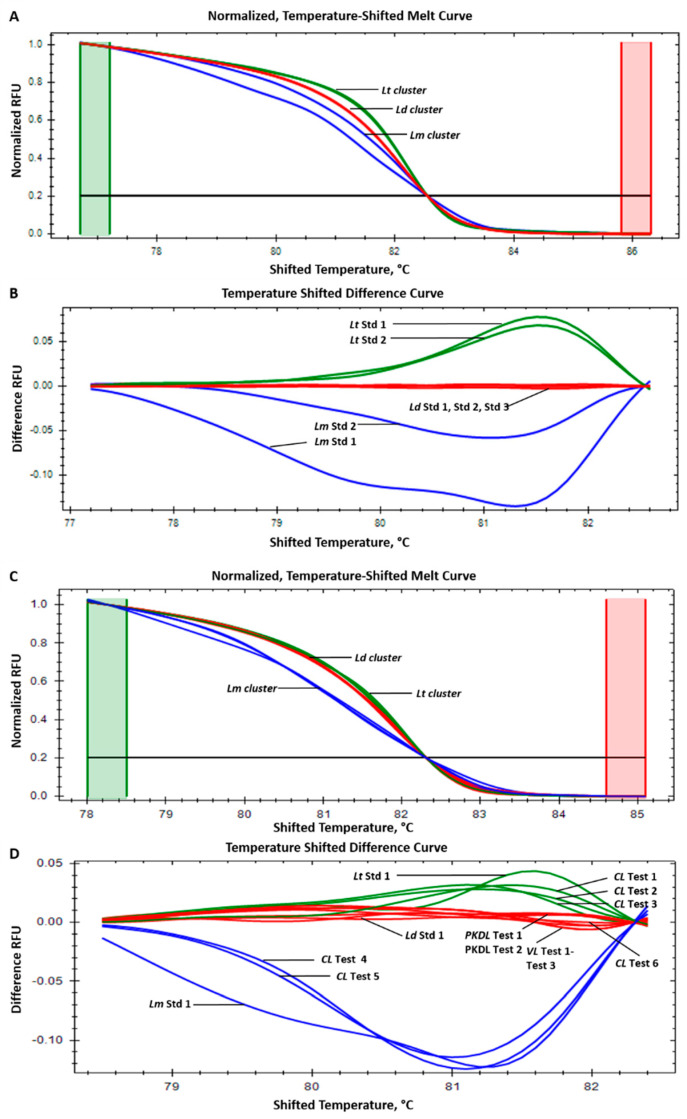
High-resolution melting (HRM) curves of the ITS-1 gene RT-PCR (qITS1sps-F1/R1) amplicons. (**A**) Normalized, temperature shifted-curves for standard (Std) Leishmania species; (**B**) temperature-shifted difference curves for standard Leishmania species; (**C**) normalized, temperature-shifted curves for VL, PKDL, and CL human patient DNA samples; (**D**) temperature-shifted difference curves for VL, PKDL, and CL human patient DNA samples. Ld Std1- *L. donovani* (MHOM/IN/95/9515); Ld Std2- *L. donovani* (MHOM/IN/1983/AG83); Ld Std3- *L. donovani* (MHOM/SD/62/1S); Lm Std1- *L. major* (MHOM/SN/74/SD); Lm Std2- *L. major* (MHOM/SU/73/5ASKH); Lt Std1- *L. tropica* (MHOM/AF/87/RUP); Lt Std2- *L. tropica* (MHOM/IL/1990/P283). VL Test1 to Test3- visceral leishmaniasis test samples; PKDL Test1 and Test2-post-kala-azar dermal leishmaniasis test samples; CL Test1 to Test6- cutaneous leishmaniasis test samples. Cluster curve representation: *Ld* cluster- *L. donovani* (red); *Lm* cluster- *L. major* (blue); *Lt* cluster- *L. tropica* (green).

**Figure 3:**
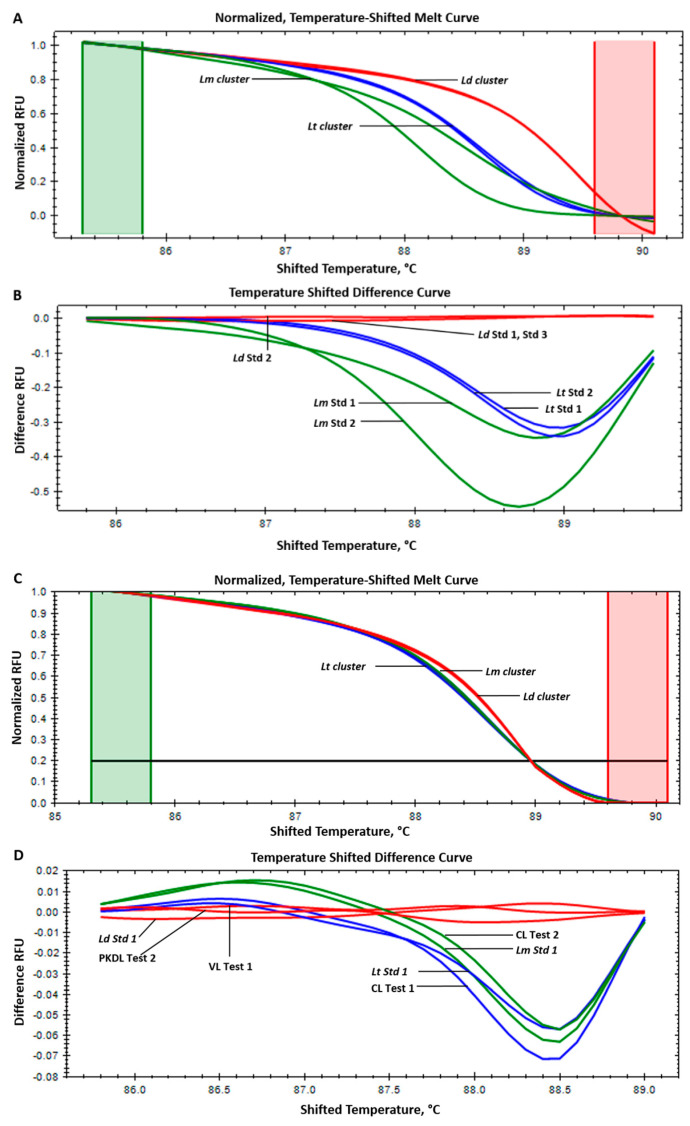
High-resolution melting (HRM) curves of the 7SL RNA gene RT-PCR amplicons. (**A**) Normalized, temperature-shifted curves for standard (Std) *Leishmania* species; (**B**) temperature-shifted difference curves for standard *Leishmania* species; (**C**) normalized, temperature-shifted curves for VL, PKDL, and CL human patient DNA samples; (**D**) temperature-shifted difference curves for VL, PKDL, and CL human patient DNA samples. Ld Std1- *L. donovani* (MHOM/IN/95/9515); Ld Std2- *L. donovani* (MHOM/IN/1983/AG83); Ld Std3- *L. donovani* (MHOM/SD/62/1S); Lm Std1- *L. major* (MHOM/SN/74/SD); Lm Std2- *L. major* (MHOM/SU/73/5ASKH); Lt Std1- *L. tropica* (MHOM/AF/87/RUP); Lt Std2- *L. tropica* (MHOM/IL/1990/P283). VL Test1- visceral leishmaniasis test samples; PKDL Test1- post-kala-azar dermal leishmaniasis test samples; CL Test1 and Test2- cutaneous leishmaniasis test samples. Cluster curve representation: *Ld* cluster- *L. donovani* (red); *Lm* cluster- *L. major* (green); *Lt* cluster- *L. tropica* (blue).

Normalized, temperature-shifted melting curves and temperature-shifted difference curves for the standard strains, i.e., *L. donovani*, *L. major*, and *L. tropica*, are shown in Figure 2A,B. The ITS-1 HRM-PCR (primer set qITS1sps-F1/R2) showed a Tm range of 82.3 ± 0.42, 82.7 ± 0.14, and 82.5 ± 0.12 for *L. donovani*, *L. major*, and *L. tropica*, respectively, with a similar overlapping melt curve. Since the primer set qITS1sps-F1/R2 produced a similar melt profile for all three standard strains, it was not used for testing patient samples. In the HRM-PCR targeting the 7SL-RNA gene, the melting temperatures (Tms) of the standard strains *L. donovani*, *L. major*, and *L. tropica* were 89.5 ± 0.35 °C, 88.07 ± 0.16 °C, and 88.5 ± 0.16 °C, respectively. Normalized, temperature-shifted melting curves and temperature-shifted difference curves for the standard strains are shown in Figure 3A,B.

### 3.2. Limit of Detection

For the ITS-1 HRM-PCR (qITS1sps-F1/R1), the assay could detect up to 0.01 pg of template DNA of *L. major* and up to 0.1 pg for *L. donovani* and *L. tropica.* For the 7SL RNA HRM-PCR, the LOD was 1 pg for *L. major* and 10 pg for *L. donovani* and *L. tropica.* PCR efficiency was calculated by selecting the Ct value points for template DNA starting from 10 ng up to LOD values for each standard strain. PCR efficiency results are given in Table 3. For the ITS-1 HRM-PCR, the efficiency ranged from 89.4 to 94.3% for three standard strains, with an average reaction efficiency of 91.4%. In the 7SL-RNA HRM-PCR, the efficiency was in a similar range from 80.5 to 96.0%, with an average of 90.4% for the assay.

With the parasite DNA load ranging from 10 ng to 1 fg, the ITS-1 amplicon showed a very steady Tm value for *L. donovani* (81.90 ± 0 to 82.40 ± 0.14), *L. major* (83.20 ± 0 to 83.40 ± 0), and *L. tropica* (82.00 ± 0 to 82.10 ± 0.5). The average Tm of the ITS-1 amplicon for the three *Leishmania* species with respect to parasite load (represented by the Ct value) is given in Table S1.

The specificity of both assays was 100%, as no amplification was noted in the HRM-PCR when using non-leishmania DNA templates from *Plasmodium falciparum*, *Mycobacterium leprae*, *Toxoplasma gondii*, *E. coli*, and non-infected healthy control samples.

To determine the specificity of the *Leishmania* ITS-1 gene-specific HRM-PCR primers (qITS1sps-F1/R1) for *Trypanosoma* spp., we performed an in silico analysis of various *Trypanosoma* spp. sequences of ITS-1. The results showed a partial reverse primer share homology, while the forward primer did not have any homology with the Trypanosoma sequences (Supplementary Figure S1).

### 3.3. Validation of HRM-PCR on Leishmaniasis Patient Samples

Of the 93 total leishmaniasis samples, ITS-1 HRM-PCR amplified in 68 samples with a sensitivity of 73.11% (Table 4). Overall, 21/30 VL samples and 37/50 PKDL patient samples presented a melting profile (Tm and melting curve) for *L. donovani*. Additionally, 10/13 CL samples had the melt profiles of *L. major* (*n* = 3), *L. tropica* (*n* = 6), and *L. donovani* (*n* = 1). The melting curves of HRM-PCR positive patient samples were grouped into three clusters specific to reference strains, enabling clear species identification. Normalized, temperature-shifted melting curves and temperature-shifted difference curves for VL, PKDL, and CL samples are shown in Figure 2C,D.

HRM-PCR targeting the 7SL-RNA gene showed amplification in 18/93 samples, presenting a sensitivity of 19.35%. The 7SL-RNA HRM-PCR showed a melting profile for 8/30 VL samples and 6/50 PKDL samples as *L. donovani* and for 4/13 CL samples as *L. major* (*n* = 2) and *L. tropica* (*n* = 2). Normalized, temperature-shifted melting curves and temperature-shifted difference curves for VL, PKDL, and CL samples are shown in Figure 3C,D.

The HRM-PCR data were also validated by sequencing the ITS-1 region (~340 bp) amplified by nested PCR as described earlier (primer information given in Table 1) [32]. The ITS-1 region was amplified in selected samples, including six PKDL (PQ157884 to PQ157889) and three CL (PQ157880, PQ157882, PQ157883) samples (with a low Ct value in the kDNA qPCR of parasite load > 10,000 parasite DNA/reaction), and sequenced. The sequence analysis data (Supplementary Table S2) corroborated the HRM PCR results.

### 3.4. Correlation of HRM-PCR Positivity with Parasite Load

**Figure 4:**
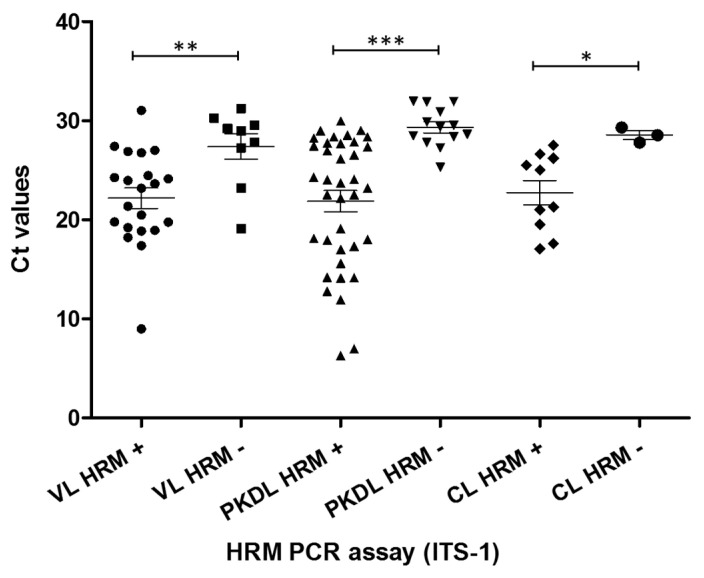
Agreement of HRM PCR amplification with parasite load in human patient samples of VL, PKDL, and CL. X-axis: patient DNA samples showing positive/negative amplification in HRM PCR (targeting ITS-1 gene). Y-axis: parasite load presented in terms of Ct value generated in kDNA qPCR. VL HRM+: visceral leishmaniasis samples having positive amplification in HRM-PCR; VL HRM-: visceral leishmaniasis samples having no amplification in HRM-PCR; PKDL HRM+: post-kala-azar dermal leishmaniasis samples having positive amplification in HRM-PCR; PKDL HRM-: post-kala-azar dermal leishmaniasis samples having no amplification in HRM-PCR; CL HRM+: cutaneous leishmaniasis samples having positive amplification in HRM-PCR; CL HRM-: cutaneous leishmaniasis samples having no amplification in HRM-PCR. *, **, ***, *p* = 0.007, *p* = 0.0002, and *p* = 0.03, respectively.

The ITS-1 HRM-PCR positive and negative samples were plotted against the Ct values generated in the kDNA qPCR (indicative of the parasite load in patient samples). A significant correlation was observed among ITS-1 HRM-PCR positive samples, which showed lower Ct values in kDNA qPCR compared to ITS-1 HRM-PCR negative samples (Figure 4). The level of significance for VL, PKDL, and CL patient samples were *p* = 0.007, *p* = 0.0002, and *p* = 0.03, respectively. However, no correlation could be established for the 7SL-RNA HRM-PCR as fewer than 20% of the samples were amplified in the assay.

## 4. Discussion

In India, the National Kala-Azar Elimination Programme (NKEP) has made significant progress in eliminating VL. In the four endemic states, the program has achieved the VL elimination target in 625 of the 633 endemic blocks, which means less than one case per 10,000 population per year at the block level [33]. This success can be attributed to the introduction of rapid diagnosis test kits, even in areas with limited transportation facilities, as well as the use of safe oral drugs like miltefosine and reducing vector density by indoor residual spraying with synthetic pyrethroids.

Now that India is close to eliminating VL, the focus should shift to eliminating CL and treating PKDL, as these act as reservoirs of the *Leishmania* parasite. The co-occurrence of VL and CL cases caused by *L. donovani*, evident in Himachal Pradesh, may serve as a reservoir for VL recurrence [34]. PKDL presents unique challenges due to its painless skin lesions, slow progression, and potential misdiagnosis as leprosy or vitiligo [5]. Therefore, rapid diagnosis and accurate identification are of utmost importance for precise prognosis and control of these diseases.

HRM-PCR has been found to be applicable in species identification and can be directly applied to clinical samples, bypassing the need for parasite isolation and in vitro culture required for other speciation methods. The sensitivity and specificity of the HRM-PCR assay to apply in a given geographic region depends on the selection of the appropriate locus to answer for regional genetic variations among the *Leishmania* species [19].

In this study, we evaluated two genetic loci, ITS-1 and 7SL-RNA, to identify different species of Old World *Leishmania*. Our findings revealed that the ITS-1 based assay was more sensitive than the 7SL-RNA based assay, as it could detect DNA from 1 parasite per microlitre. In an earlier study that targeted the ITS-1 region, 0.05 pg of DNA was detected using HRM-PCR from Old World cultured promastigotes [22]. The ITS-1 based assay successfully identified species in over 70% of clinical samples, whereas the 7SL-RNA based assay could only determine species in less than 20% of clinical samples. The efficacy of the assay was dependent on the amount of parasite present in the patients, as indicated by the significant correlation observed in the kDNA based Ct values and HRM-PCR positivity in the clinical sample. Other researchers have also observed the low positivity of the 7SL-RNA based assay, possibly due to the low target available in the clinical material or the assay conditions [25]. We attempted the assay under previously defined conditions, but the target could not be amplified. With conditions optimized in this study for the 7SL-RNA based assay, we still observed a low amplification rate in clinical samples, and the LOD for this assay was 100 times lower than the ITS-1 based assay. The results of this study suggest that the ITS-1 based HRM-PCR is more sensitive and suitable for detecting parasite species circulating in India. The ITS-1 DNA region exhibits a high degree of conservation among various species of trypanosomatid parasites [35]. Even with small variations in the melting temperatures of similar sequences, the ITS-1 locus could accurately differentiate between species, allowing for the simultaneous processing of multiple samples from various manifestations of leishmaniasis seen in India.

The results of HRM analysis are concluded on the basis of Tm and melting curve. Multiple factors, such as GC content, sequence read, and amplicon, are fundamental to variations in Tm and determine the melting curve pattern [36]. The Tm positively correlates with sequence length and percent GC content (%G + C). For smaller DNA fragments, single-base-pair changes in the %G + C content typically have predictable effects that are consistently observed in the Tm. In the case of the ITS-1 target, the amplicon had distinct Tm values for *L. donovani*, *L. major*, and *L. tropica*. Table 3 shows the observed Tm range for both standard strains and clinical samples that were evaluated in this study. Genetic changes, such as single nucleotide polymorphisms (SNPs) or insertions/deletions (indels), will directly affect the melting profile (melting curve or Tm or both) of a DNA sequence. Additionally, the slight overlap in the range of Tm can be attributed to the initial template DNA concentration in the patient samples, as observed earlier [21].

HRM analysis also makes use of the precise melting curve shape, which is a function of the DNA sequence [36]. For example, if regions of the sequence have different %GC contents, this can result in bi- or multiphasic melting curves, which can assist in sequence discrimination. Temperature-shifted melt curves enhance the visualization of the presence of heteroduplex and homoduplex DNA. In this study, the *Leishmania* ITS-1 gene-specific HRM-PCR primers (qITS1sps-F1/R1) give very distinct melt curves for *L. donovani*, L. major, and L. tropica spp. The data were complemented with three distinct curves obtained in both normalized temperature-shifted melt curves and when observed in a temperature-shifted difference curve format (Figure 2). Discrepancies in discriminating the Leishmania species arising from overlapping Tm values among clinical samples can be resolved by analyzing the melt curve pattern. However, the qITS1sps-F1/R1-based HRM analysis may have limitations in identifying *L. tropica* or *L. major* with SNPs or indels that affect the melting profile, as these may generate a distinct Tm or the shape of the melt curve reported in this study.

For the 7SL-RNA gene amplicon, the Tm was 89.5 ± 0.35 for *L. donovani* but in a very close range for *L. major* and *L. tropica* (88.07 ± 0.16 and 88.5 ± 0.16). Moreover, a very similar pattern melt curves for *L. major* and *L. tropica* makes them indistinguishable (both in the normalized temperature-shifted melt curve and temperature-shifted curve difference formats). These results make *L. major* and *L. tropica* discrimination challenging.

An assay that can identify the causative *Leishmania* species is critical for the pharmaceutical selection and decision of a species-selective regimen. In the context of VL and CL co-inhibiting areas and atypical dermal cases of PKDL and CL, the identification of the causative *Leishmania* spp. would facilitate easy categorization. The HRM-PCR, with its high sensitivity, is a robust tool in the identification of natural leishmania infection, particularly in field sampling. Using the ITS-1 HRM-PCR assay, the presence of *L. donovani*, *L. major*, and *L. tropica* was successfully detected with remarkable sensitivity, allowing for the identification of as little as 1 parasite DNA per reaction. The HRM-PCR assay developed in this study has proven its efficiency in identifying natural *Leishmania* infection samples with different manifestations in tertiary care hospitals serving patients from both endemic and non-endemic regions. Thus, this assay provides a reliable method for identifying *Leishmania* infection, instilling confidence in its potential application in endemic areas.

## 5. Conclusions

In conclusion, the diverse clinical manifestations of leishmaniasis and the genetic variations among *Leishmania* species necessitate the development and implementation of accurate and cost-effective molecular techniques for species identification. The use of advanced molecular methods such as HRM analysis holds promise for improving the accuracy and efficiency of *Leishmania* species identification. The ITS-1 HRM-PCR assay could identify *Leishmania* spp. causing different clinical forms of leishmaniasis in the Indian subcontinent, providing rapid and accurate results that can guide clinical management and treatment decisions.

## Figures and Tables

**Table 1:** List of standard *Leishmania* strains used for the establishment of the HRM assay.

Isolate Code	Isolate	Strain	Accession No.
Ld Std1	*L. donovani*	MHOM/IN/95/9515	PQ157709
Ld Std2	*L. donovani*	MHOM/IN/1983/AG83	OQ740303
Ld Std3	*L. donovani*	MHOM/SD/62/1S	PQ157708
Lm Std1	*L. major*	MHOM/SN/74/SD	PQ157881
Lm Std2	*L. major*	MHOM/SU/73/5ASKH	KU680845
Lt Std1	*L. tropica*	MHOM/AF/87/RUP	PQ157674
Lt Std2	*L. tropica*	MHOM/IL/1990/P283	MZ366760

Ld—*L. donovani*; Lm—*L. major*; Lt—*L. tropica*; Std—standard strain.

**Table 2:** List of oligo sequences used in this study for the molecular confirmation of leishmaniasis and identification of *Leishmania* spp.

PCR Technique	Target	Primer	Oligo Sequence	Amplicon Size (bp)	Reference
HRM-PCR	Internal Transcribed Spacer- 1 rRNA gene	qITS1sps-F1	5′-GCCTTTCCCACATACACAGC-3′	Ld-184 Lm-201 Lt-178	This study
		qITS1sps-R1	5′-TCGCGACACGTTATGTGAG-3′		
		qITS1sps-R2	5′-AAGCCAAGTCATCCATCGC-3′		
	7SL-RNA gene	7SL-RNA F1	5′-ACGTGGACCAGCGAGGGT-3′	Ld-119 Lm-118 Lt-118	[30]
		7SL-RNA R1	5′-CGGTTCCCTCGCTTCAAC-3′		
Nested PCR	Internal Transcribed Spacer- 1 rRNA gene	ITS Full F1	5′-CTGGATCATTTTCCGATG-3′	~1000–1100	[32]
		ITS Full R2	5′-CTCTCTTTTTTCTCTGTGC-3′		
		ITS1-F1	5′-CTGGATCATTTTCCGATG-3′	Ld-319 Lm-338 Lt-321	
		ITS1-R1	5′-TGATACCACTTATCGCACTT-3′		
qPCR	kDNA	QT1-F	5′-CTTTTCTGGTCCTCCGGGTAGG-3′	210bp	[26]
		QT1-R	5′-CCACCCGGCCCTATTTTACACCAA-3′		

Ld—*L. donovani*; Lm—*L. major*; Lt—*L. tropica*.

**Table 3:** Average melting temperature (Tm) value of dissociation curves, limit of detection (LOD), and PCR efficiency for HRM-PCR. The average Tm is calculated for standard strains and human patient DNA samples tested in this study (run in duplicate). PCR efficiency calculations were made within the LOD for each amplicon.

*Leishmania* spp.	ITS-1 Gene				7SL RNA Gene		
	qITS1sps-F1/R1			qITS1sps-F1/R2	7SL-RNA F1/R1		
	Tm (Mean ± SD) (°C)	LOD	PCR Efficiency	Tm (Mean ± SD) (°C)	Tm (Mean ± SD) (°C)	LOD	PCR Efficiency
*L. donovani*	81.82 ± 0.46	0.1 pg	89.8%	82.3 ± 0.42	89.5 ± 0.35	10 pg	80.5%
*L. major*	83.3 ± 0.70	0.01 pg	90.1%	82.7 ± 0.14	88.07 ± 0.16	1 pg	96.0%
*L. tropica*	82.4 ± 0.51	0.1 pg	94.3%	82.5 ± 0.12	88.5 ± 0.16	10 pg	94.8%

**Table 4:** *Leishmania*-infected human patient samples tested for species identification using HRM-PCR.

Clinical Manifestation	Samples Tested	High Resolution Melt Curve Analysis			
		ITS-1 (Species Identified) and %Positivity		7SL RNA (Species Identified) and %Positivity	
VL	30	*L. donovani* (*n* = 21)	21 (70%)	*L. donovani* (*n* = 8)	8 (26.6%)
PKDL	50	*L. donovani* (*n* = 37)	37 (74%)	*L. donovani* (*n* = 6)	6 (12%)
CL	13	*L. major* (*n* = 3) *L. tropica* (*n* = 6) *L. donovani* (*n* = 1)	10 (76.9%)	*L. major* (*n* = 2) *L. tropica* (*n* = 2)	4 (30.7%)

## Data Availability

The authors confirms that all the reported data are available in the manuscript.

## Supplementary Materials

The following supporting information can be downloaded at: https://www.mdpi.com/article/10.3390/pathogens13090759/s1. Table S1: Mean Ct values and melting temperature (Tm) values of dissociation curves for Leishmania standard strain sample DNA dilution series generated in HRM PCR assay (primers- qITS1sps-F1/R1). Table S2. Nucleotide sequences of ITS- region amplified and sequenced from PKDL and CL human patient samples. Figure S1: Multiple alignment of ITS-1 sequences of *Trypanosoma* spp. (*T. cruzi* (AF362825), *T. evansi* (MN121259), *T. equiperdum* (LC386049), *T. brucei* (MW364108)). File S1: ITS sequences multiple alignment for primer design.
