# Supplementary material for: Assessment of High-Resolution Melting Curve Analysis for Leishmania spp. Detection in Different Clinical Manifestations of Leishmaniasis in India
Source: Pathogens. 2024 Sep 4;13(9):759. doi: 10.3390/pathogens13090759 (PMC11435223; doi:10.3390/pathogens13090759)

Table S1: Mean Ct values and melting temperature (Tm) values of dissociation curves for Leishmania standard strain sample DNA dilution series generated in HRM PCR assay (primers- qITS1sps-F1/R1) .

| **Leishmania strain- DNA dilution series** | ***L. donovani (MHOM/IN/95/9515)*** | | ***L. major (MHOM/SN/74/SD)*** | | ***L. tropica (MHOM/AF/87/RUP)*** | |
| --- | --- | --- | --- | --- | --- | --- |
|  | **Ct**  **(mean ±SD)** | **Tm**  **(mean ±SD)** | **Ct**  **(mean ±SD)** | **Tm**  **(mean ±SD)** | **Ct**  **(mean ±SD)** | **Tm**  **(mean ±SD)** |
| 10 ng | 16.86 ±0.11 | 81.90 ±0 | 15.52 ±0.04 | 83.30 ±0.14 | 16.06 ±0.02 | 82.00 ±0 |
| 1 ng | 20.41 ±0.02 | 81.90 ±0 | 19.26 ±0.01 | 83.30 ±0.14 | 19.23 ±0.08 | 82.00 ±0 |
| 100pg | 24.24 ±0 | 82.00 ±0.14 | 23.11 ±0.08 | 83.20 ±0 | 22.99 ±0.12 | 82.00 ±0 |
| 10 pg | 28.17 ±0.23 | 82.10 ±0 | 26.58 ±0.02 | 83.30 ±0.14 | 26.92 ±0.11 | 82.00 ±0 |
| 1 pg | 32.18 ±0.11 | 82.30 ±0 | 30.71 ±0 | 83.30 ±0.14 | 30.26 ±0.06 | 82.10 ±02 |
| 100 fg | 34.70 ±1.88 | 82.40 ±0.14 | 33.62 ±0.25 | 83.40 ±0 | 33.55 ±0.30 | 82.10 ±05 |
| 10 fg | N/A | None | 36.61 ±0.28 | 83.40 ±0 | 36.85 ±0.57 | None |
| 1 fg | N/A | None | N/A | None | N/A | None |

N/A- not amplified None- no melt curve detected.

Table S2. Nucleotide sequences of ITS- region amplified and sequenced from PKDL and CL human patient samples.

| **Leishmaniasis** | **Patient sample** | **Accession no.** | **Leishmania *sps*** | **Nucleotide sequence** |
| --- | --- | --- | --- | --- |
| PKDL | Case1-PB8 | PQ157884 | *L. donovani* | CTGGATCATTTTCCGATGATTACACCAAAAAAAACATATACAACTCGGGGAGACCTATGTATATATATATGTAGGCCTTTCCCACATACACAGCAAAGTTTTGTACTCAAAATTTGCAGTAAAAAAAGGCCGATCGACGTTATAACGCACCGCCTATACAAAAGCAAAAATGTCCGTTTATACAAAAAATATACGGCGTTTCGGTTTTTGGCGGGGTGGGTGCGTGTGTGGATAACGGCTCACATAACGTGTCGCGATGGATGACTTGGCTTCCTATTTCGTTGAAGAACGCAGTAAAGTGCGATAAGTGGTATCA |
|  | Case2- PB26 | PQ157885 | *L. donovani* | CTGGATCATTTTCCGATGATTACACCAAAAAAAACATATACAACTCGGGGAGACCTATGTATATATATATGTAGGCCTTTCCCACATACACAGCAAAGTTTTGTACTCAAAATTTGCAGTAAAAAAAGGCCGATCGACGTTATAACGCACCGCCTATACAAAAGCAAAAATGTCCGTTTATACAAAAAATATACGGCGTTTCGGTTTTTGGCGGGGTGGGTGCGTGTGTGGATAACGGCTCACATAACGTGTCGCGATGGATGACTTGGCTTCCTATTTCGTTGAAGAACGCAGTAAAGTGCGATAAGTGGTATCA |
|  | Case3- PB35 | PQ157886 | *L. donovani* | CTGGATCATTTTCCGATGATTACACCAAAAAAAACATATACAACTCGGGGAGACCTATGTATATATATATGTAGGCCTTTCCCACATACACAGCAAAGTTTTGTACTCAAAATTTGCAGTAAAAAAAGGCCGATCGACGTTATAACGCACCGCCTATACAAAAGCAAAAATGTCCGTTTATACAAAAAATATACGGCGTTTCGGTTTTTGGCGGGGTGGGTGCGTGTGTGGATAACGGCTCACATAACGTGTCGCGATGGATGACTTGGCTTCCTATTTCGTTGAAGAACGCAGTAAAGTGCGATAAGTGGTATCA |
|  | Case4- PB60 | PQ157887 | *L. donovani* | CTGGATCATTTTCCGATGATTACACCAAAAAAAACATATACAACTCGGGGAGACCTATGTATATATATATGTAGGCCTTTCCCACATACACAGCAAAGTTTTGTACTCAAAATTTGCAGTAAAAAAAGGCCGATCGACGTTATAACGCACCGCCTATACAAAAGCAAAAATGTCCGTTTATACAAAAAATATACGGCGTTTCGGTTTTTGGCGGGGTGGGTGCGTGTGTGGATAACGGCTCACATAACGTGTCGCGATGGATGACTTGGCTTCCTATTTCGTTGAAGAACGCAGTAAAGTGCGATAAGTGGTATCA |
|  | Case5- PB56 | PQ157888 | *L. donovani* | CTGGATCATTTTCCGATGATTACACCAAAAAAAACATATACAACTCGGGGAGACCTATGTATATATATATGTAGGCCTTTCCCACATACACAGCAAAGTTTTGTACTCAAAATTTGCAGTAAAAAAAGGCCGATCGACGTTATAACGCACCGCCTATACAAAAGCAAAAATGTCCGTTTATACAAAAAATATACGGCGTTTCGGTTTTTGGCGGGGTGGGTGCGTGTGTGGATAACGGCTCACATAACGTGTCGCGATGGATGACTTGGCTTCCTATTTCGTTGAAGAACGCAGTAAAGTGCGATAAGTGGTATCA |
|  | Case6- PK108 | PQ157889 | *L. donovani* | CTGGATCATTTTCCGATGATTACACCAAAAAAAACATATACAACTCGGGGAGACCTATGTATATATATATGTAGGCCTTTCCCACATACACAGCAAAGTTTTGTACTCAAAATTTGCAGTAAAAAAAGGCCGATCGACGTTATAACGCACCGCCTATACAAAAGCAAAAATGTCCGTTTATACAAAAAATATACGGCGTTTCGGTTTTTGGCGGGGTGGGTGCGTGTGTGGATAACGGCTCACATAACGTGTCGCGATGGATGACTTGGCTTCCTATTTCGTTGAAGAACGCAGTAAAGTGCGATAAGTGGTATCA |
| CL | Case1-  CL27 | PQ157880 | *L. major* | CTGGATCATTTTCCGATGATTACACCCCAAAAAACATATACAACTCGGGGAGGCTTATTCTATATATATATAGTATAGGCTTTTCCCACATACACAGCAAACTTTTATACTCGAAATTTGCAGTAAAAAAGGCCGATCGACGTTGTAGAACGCACCGCCTATACACAAAAGCAAAAATGTCCGTTTATACAAAAAAATAGACGGCGTTTCGGTTTTTGGCGGGAGGGAGAGAGAGGGGGGTGCGTGCGCGTGGATAACGGCTCACATAACGTGTCGCGATGGATGACTTGGCTTCCTATTTCGTTGAAGAACGCAGTAAAGTGCGATAAGTGGTATCA |
|  | Case2-CL33 | PQ157882 | *L. tropica* | CTGGATCATTTTCCGATGATTACACCCCCAAAAAAAAACATATACAAAACTCGGGGAGGCCTATTTATATACATTATAGGCCTTTCCCACATACACAGCAAACTTTTATACTCGAAGTTTGCAGTAAACAAAAGGCCGATCGACGTTATAACGCACCGCCTATACACAAAAGCAAAAATGTCCGTTTATACAAATATACGGCGTTTCGGTTTTGTTGGCGGGGGGTGCGTGTGTGGATAACGGCTCACATAACGTGTCGCGATGGATGACTTGGCTTCCTATTTCGTTGAAGAACGCAGTAAAGTGCGATAAGTGGTATCA |
|  | Case3-CL37 | PQ157883 | *L. tropica* | CTGGATCATTTTCCGATGATTACACCCCCAAAAAAAAACATATACAAAACTCGGGGAGGCCTATTTATATACATTATAGGCCTTTCCCACATACACAGCAAACTTTTATACTCGAAGTTTGCAGTAAACAAAAGGCCGATCGACGTTATAACGCACCGCCTATACACAAAAGCAAAAATGTCCGTTTATACAAATATACGGCGTTTCGGTTTTGTTGGCGGGGGGTGCGTGTGTGGATAACGGCTCACATAACGTGTCGCGATGGATGACTTGGCTTCCTATTTCGTTGAAGAACGCAGTAAAGTGCGATAAGTGGTATCA |

Figure S1: Multiple alignment of ITS-1 sequences of *Trypanosoma spp.* (*T. cruzi* (AF362825), *T. evansi* (MN121259), *T. equiperdum* (LC386049), *T. brucei* (MW364108)).


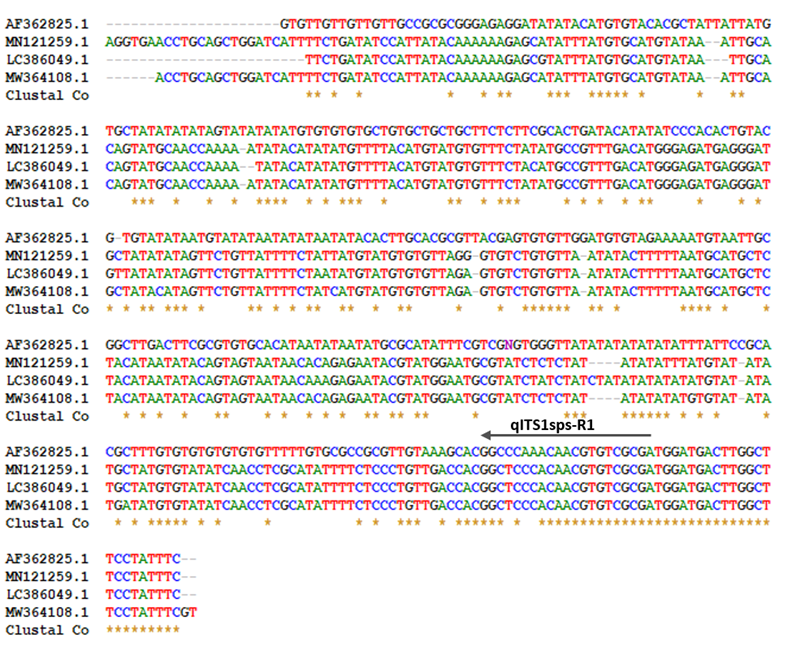

Supplement: Supplementary file 1 [file pathogens-13-00759-s001.zip › pathogens-3042570-supplementary/Supplementary Table S1.docx]
